# Supplementary material for: Higher Expressions of SHH and AR Are Associated with a Positive Receptor Status and Have Impact on Survival in a Cohort of Croatian Breast Cancer Patients
Source: Life (Basel). 2022 Oct 7;12(10):1559. doi: 10.3390/life12101559 (PMC9605052; doi:10.3390/life12101559)
Supplement: Supplementary file 1 [file life-12-01559-s001.zip › life-1936520-supplementary/Supplemental.Figure.S1.pptx]

## Slide 1
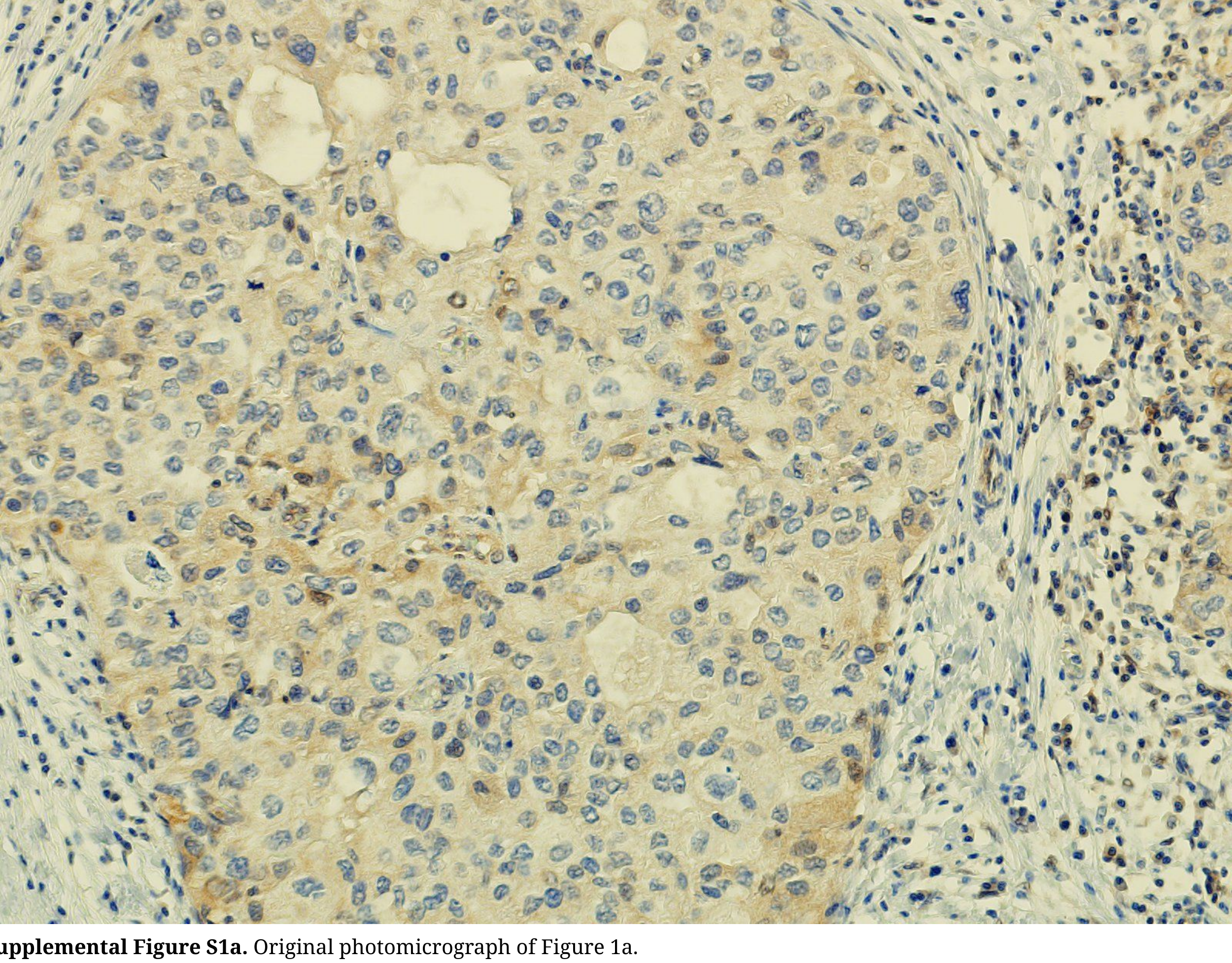

Supplemental Figure S1a. Original photomicrograph of Figure 1a.

## Slide 2
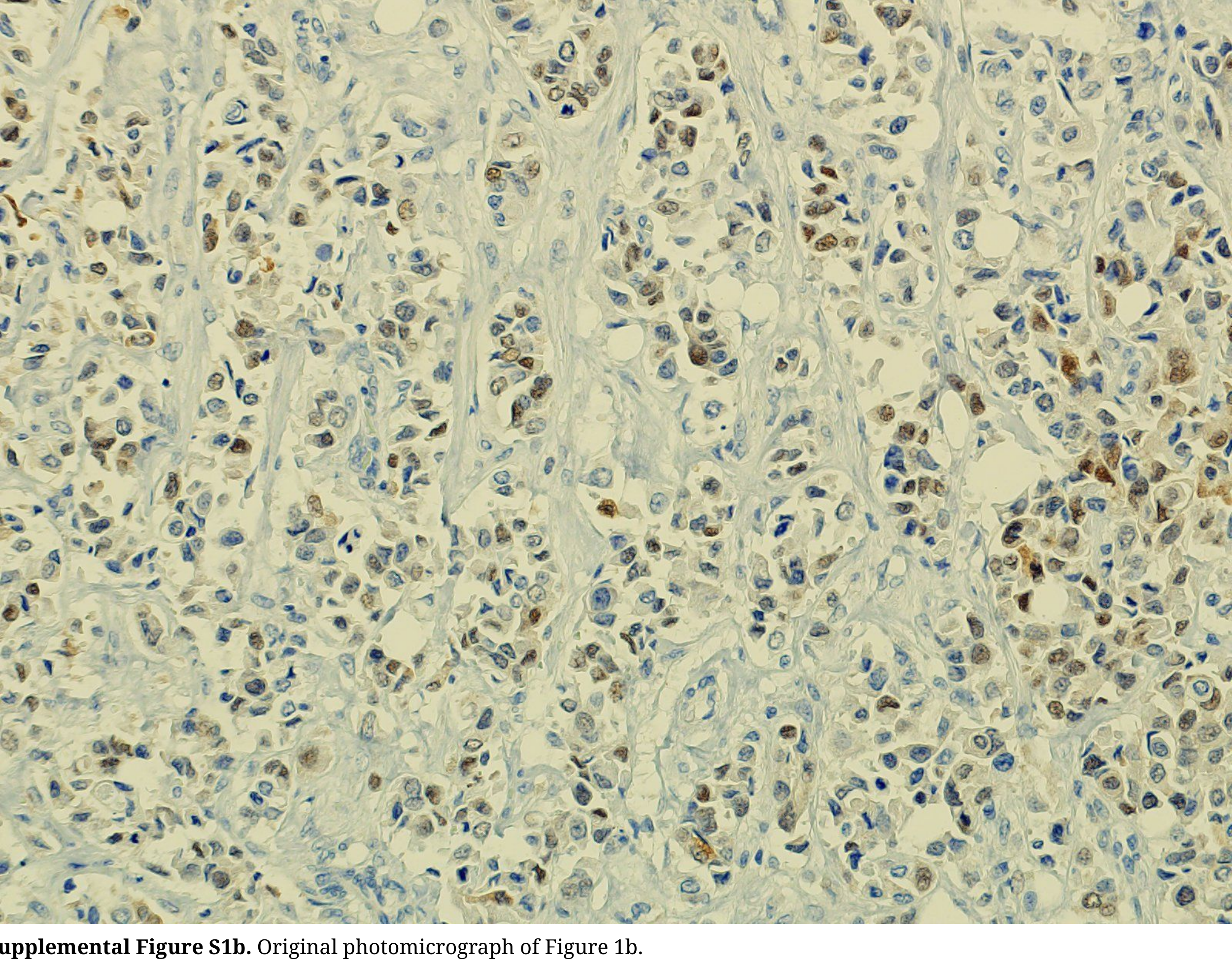

Supplemental Figure S1b. Original photomicrograph of Figure 1b.

## Slide 3
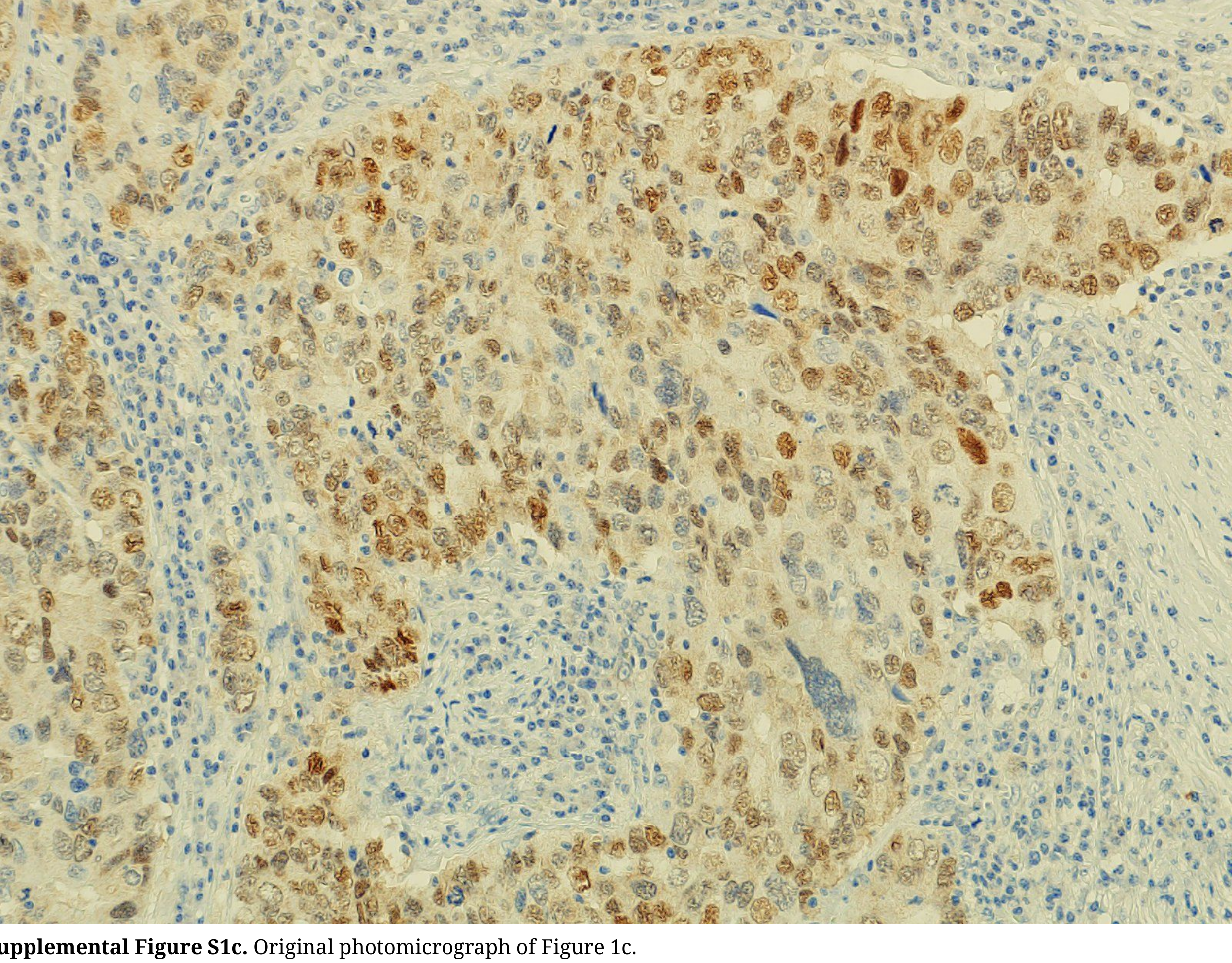

Supplemental Figure S1c. Original photomicrograph of Figure 1c.

## Slide 4
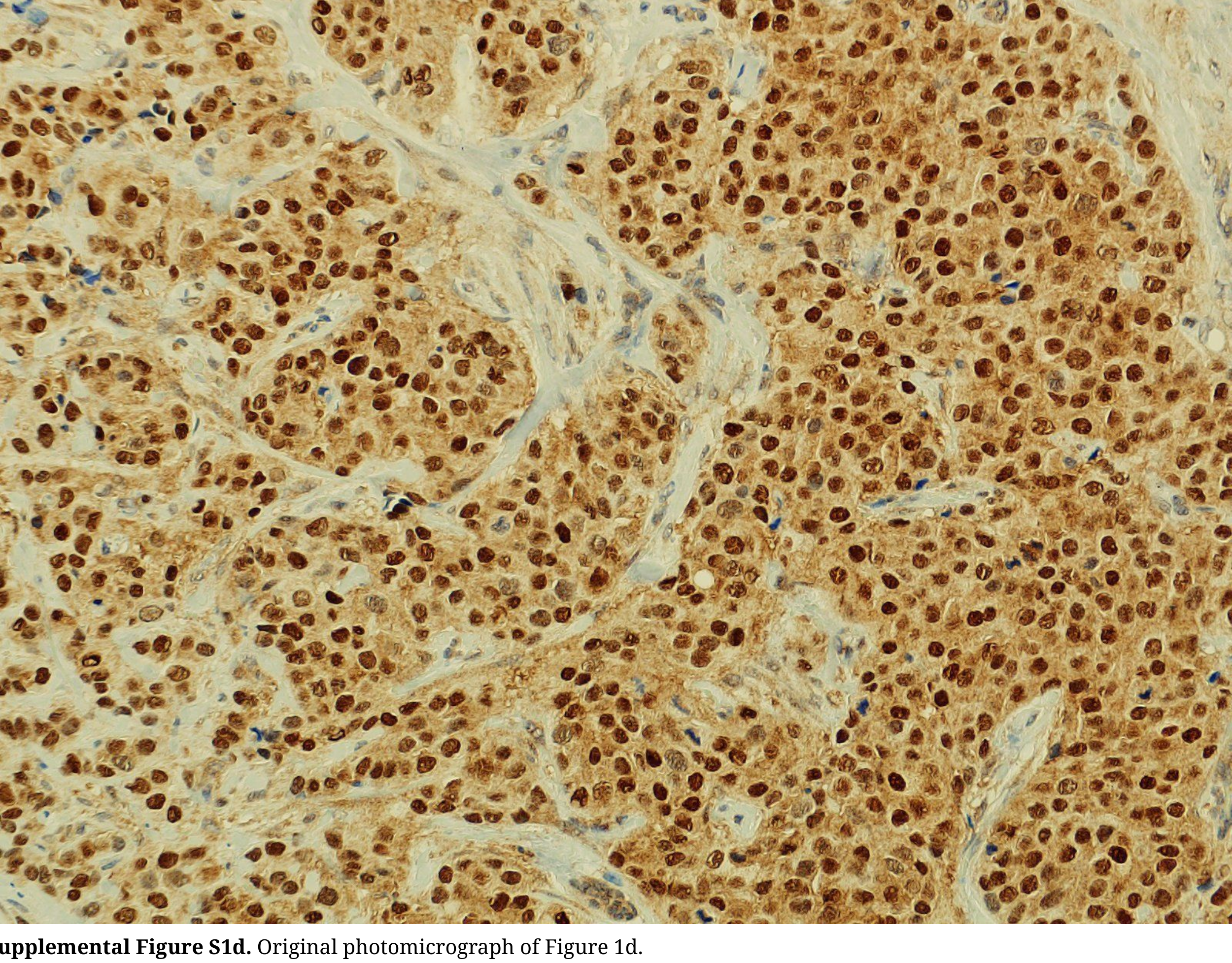

Supplemental Figure S1d. Original photomicrograph of Figure 1d.
