# Supplementary material for: Higher Expressions of SHH and AR Are Associated with a Positive Receptor Status and Have Impact on Survival in a Cohort of Croatian Breast Cancer Patients
Source: Life (Basel). 2022 Oct 7;12(10):1559. doi: 10.3390/life12101559 (PMC9605052; doi:10.3390/life12101559)
Supplement: Supplementary file 1 [file life-12-01559-s001.zip › life-1936520-supplementary/Supplemental.Tables.S1-S4.pdf]

Supplemental Table S1. Association of clinicopathological characteristics of a cohort of Croatian breast cancer patients with molecular subtype and receptor status. Statistically significant *p*-values for chi-square or Fisher's exact test are in bold. Data are presented with a number of samples (percentage of the whole column).

**Characteristic**	**Molecular subtype**					**Receptor status**								
Age	**LumA**	**LumB**	**HER2-E**	**TNBC**	***p***	**ER-**	**ER+**	***p***	**PR-**	**PR+**	***p***	**HER2-**	**HER2+**	***p***
≤50 years	7 (14.6)	22 (30.1)	2 (12.5)	15 (31.9)	0.096	17 (26.6)	29 (24.2)	0.722	21 (26.6)	25 (23.8)	0.668	34 (24.1)	12 (27.9)	0.616
>50 years	41 (85.4)	51 (69.9)	14 (87.5)	32 (68.1)		47 (73.4)	91 (75.8)		58 (73.4)	80 (76.2)		107 (75.9)	31 (72.1)	
Histotype														
IDC	42 (87.5)	55 (74.3)	16 (100.0)	47 (100.0)	**0.0002**	64 (100.0)	96 (79.3)	**0.0001**	76 (96.2)	84 (79.2)	**0.0009**	123 (86.6)	37 (86.0)	0.924
IDC + DCIS	6 (12.5)	19 (25.7)	0 (0.0)	0 (0.0)		0 (0.0)	25 (20.7)		3 (3.8)	22 (20.8)		19 (13.4)	6 (14.0)	
Tumor size														
<2 cm	38 (79.2)	43 (58.1)	7 (43.7)	17 (36.2)	**0.0003**	25 (39.1)	80 (66.1)	**0.0004**	37 (46.8)	68 (64.2)	**0.019**	89 (62.7)	16 (37.2)	**0.003**
≥2 cm	10 (20.8)	31 (41.9)	9 (56.2)	30 (63.8)		39 (60.9)	41 (33.9)		42 (53.2)	38 (35.8)		53 (37.3)	27 (62.8)	
Nodal involvement														
0-3	46 (95.8)	60 (82.2)	12 (75.0)	41 (87.2)	0.088	54 (84.4)	105 (87.5)	0.557	65 (82.3)	94 (89.5)	0.157	128 (90.8)	31 (72.1)	**0.002**
≥4	2 (4.2)	13 (17.8)	4 (25.0)	6 (12.8)		10 (15.6)	15 (12.5)		14 (17.7)	11 (10.5)		13 (9.2)	12 (27.9)	
Ki-67														
<20%	48 (100.0)	0 (0.0)	2 (12.5)	3 (6.4)	**<0.0001**	5 (7.8)	48 (39.7)	**<0.0001**	12 (15.2)	41 (38.7)	**0.0005**	50 (35.2)	3 (7.0)	**0.0003**
≥20%	0 (0.0)	74 (100.0)	14 (87.5)	44 (93.6)		59 (92.2)	73 (60.3)		67 (84.8)	65 (61.3)		92 (64.8)	40 (93.0)	
LVI														
Absent	36 (75.0)	37 (50.0)	11 (68.7)	32 (68.1)	**0.029**	44 (68.7)	72 (59.5)	0.217	55 (69.6)	61 (57.5)	0.094	93 (65.5)	23 (53.5)	0.155
Present	12 (25.0)	37 (50.0)	5 (31.2)	15 (31.9)		20 (31.2)	49 (40.5)		24 (30.4)	45 (42.5)		49 (34.5)	20 (46.5)	
Survival														
Alive	36 (75.0)	62 (83.8)	11 (68.7)	31 (66.0)	0.140	43 (67.2)	97 (80.2)	0.051	55 (69.6)	85 (80.2)	0.098	106 (74.6)	34 (79.1)	0.555
Deceased	12 (25.0)	12 (16.2)	5 (31.2)	16 (34.0)		21 (32.8)	24 (19.8)		24 (30.4)	21 (19.8)		36 (25.4)	9 (20.9)	
Recurrence														
Absent	47 (97.9)	73 (98.6)	15 (93.7)	42 (89.4)	0.077	58 (90.6)	119 (98.3)	**0.014**	73 (92.4)	104 (98.1)	0.060	135 (95.1)	42 (97.7)	0.463
Present	1 (2.1)	1 (1.4)	1 (6.2)	5 (10.6)		6 (9.4)	2 (1.7)		6 (7.6)	2 (1.9)		7 (4.9)	1 (2.3)	
Metastasis														
Absent	48 (100.0)	68 (91.9)	14 (87.5)	36 (76.6)	**0.002**	51 (79.7)	115 (95.0)	**0.001**	65 (82.3)	101 (95.3)	**0.004**	127 (89.4)	39 (90.7)	0.812
Present	0 (0.0)	6 (8.1)	2 (12.5)	11 (23.4)		13 (20.3)	6 (5.0)		14 (17.7)	5 (4.7)		15 (10.6)	4 (9.3)	

DCIS – ductal carcinoma in situ; HER-2-E – HER-2-enriched; IDS – invasive ductal carcinoma; LumA – luminal A; LumB – luminal B; LVI – lymphovascular invasion; *p* – *p*-value; TNBC – triple-negative breast cancer

Supplemental Table S2. Impact of clinicopathological characteristics and sonic hedgehog protein and androgen receptor expressions on different types of survival in a cohort of Croatian breast cancer patients. Presented are *p*-values for log-rank test (statistically significant are in bold).

**Characteristic**		**Molecular subtype**					**Receptor status**					
		**All**	**LumA**	**LumB**	**HER2-E**	**TNBC**	**ER-**	**ER+**	**PR-**	**PR+**	**HER2-**	**HER2+**
Age<br>≤50 vs. >50 years	OS	**0.007**	0.479	0.263	0.364	**0.013**	**0.016**	0.160	**0.037**	0.093	0.052	**0.046**
	RFS	0.497	**0.022**	0.480	0.695	0.905	0.859	0.440	0.819	0.441	0.298	0.513
	MFS	0.300	N.A.	0.925	0.578	0.076	0.098	0.635	0.269	0.775	0.660	0.195
Histotype<br>IDC vs. IDC + DCIS	OS	0.095	0.236	0.493	N.A.	N.A.	N.A.	0.201	0.327	0.269	0.231	0.223
	RFS	0.250	0.691	0.545	N.A.	N.A.	N.A.	0.451	0.607	0.451	0.289	0.674
	MFS	0.077	N.A.	N.A.	N.A.	N.A.	N.A.	0.221	0.424	0.264	0.120	0.404
T<br><2 vs. ≥2 cm	OS	**0.001**	0.236	0.054	0.860	**0.030**	**0.029**	0.054	**0.010**	0.085	**0.0004**	0.293
	RFS	0.193	0.588	0.440	0.350	0.386	0.203	0.334	0.091	0.308	0.192	0.414
	MFS	**0.0006**	N.A.	0.657	0.183	**0.038**	**0.010**	0.353	**0.006**	0.240	**0.001**	0.106
N<br>0-3 vs. ≥4	OS	**0.001**	0.552	**0.014**	0.268	**0.026**	**0.026**	**0.029**	**0.006**	0.164	**0.0001**	0.188
	RFS	0.934	0.827	0.673	0.617	0.476	0.802	0.625	0.884	0.645	0.479	0.564
	MFS	**<0.0001**	N.A.	**0.028**	0.369	**0.004**	**0.006**	**0.003**	**0.002**	**0.029**	**<0.0001**	0.274
Ki-67<br><20% vs. ≥20%	OS	0.899	N.A.	N.A.	0.679	0.915	0.936	0.541	0.476	0.367	0.537	0.740
	RFS	0.258	N.A.	N.A.	0.695	0.522	0.434	0.796	0.257	0.761	0.186	0.776
	MFS	**0.016**	N.A.	N.A.	0.578	0.765	0.918	**0.034**	0.327	0.058	**0.012**	0.571
LVI<br>no vs. yes	OS	0.214	**0.040**	0.512	0.355	0.896	0.702	0.095	0.578	0.120	0.547	0.115
	RFS	0.931	0.588	0.296	0.097	0.512	0.903	0.743	0.814	0.797	0.745	0.245
	MFS	0.311	N.A.	0.398	0.445	0.804	0.516	0.164	0.587	0.078	0.675	0.204
SHH<br>'low' vs. 'high'	OS	0.232	0.374	0.162	**0.043**	0.074	**0.044**	0.259	0.084	0.097	0.095	0.608
	RFS	**0.022**	N.A.	N.A.	N.A.	**0.024**	0.131	0.360	**0.024**	0.361	**0.012**	N.A.
	MFS	0.187	N.A.	0.069	0.063	**0.042**	0.059	**0.044**	**0.044**	0.111	0.294	0.515
AR<br>'low' vs. 'high'	OS	0.193	0.171	0.079	0.110	**0.006**	**0.022**	0.202	**0.040**	0.212	0.075	**0.012**
	RFS	**0.001**	N.A.	N.A.	N.A.	0.244	0.122	**0.045**	**0.029**	**0.042**	**0.015**	N.A.
	MFS	**0.018**	N.A.	0.237	0.128	**0.008**	**0.024**	0.163	**0.044**	0.086	0.113	**0.034**

AR – androgen receptor; DCIS – ductal carcinoma *in situ*; ER – estrogen receptor; HER-2-E – HER-2-enriched; IDS – invasive ductal carcinoma; LumA – luminal A; LumB – luminal B; LVI – lymphovascular invasion; MFS – metastasis-free survival; N – number of positive lymph nodes; N.A. – no or not enough patients with endpoint or characteristic; OR – overall survival; PR – progesterone receptor; RFS – recurrence-free survival; SHH – sonic hedgehog protein; T – tumor size; TNBC – triple-negative breast cancer

Supplemental Table S3. Cox proportional hazards regression model analysis of overall survival. Multivariate analysis was performed only if SHH or AR expression showed statistically significant *p*-value in univariate analysis. Significant *p*-values are in bold.

		TNBC				ER-		PR-	
		Univariate		Multivariate		Univariate		Univariate	
**Covariate**	**Categories**	**HR (95% CI)**	***p***	**HR (95% CI)**	***p***	**HR (95% CI)**	***p***	**HR (95% CI)**	***p***
Age	≤50 vs. >50 years	8.5 (1.12–64.61)	**0.038**	9.4 (1.23–71.30)	**0.031**	8.0 (1.07–59.84)	**0.042**	4.1 (0.97–17.66)	0.055
T	<2 vs. ≥2 cm	4.5 (1.02–19.77)	**0.047**	-(-)	-	3.2 (1.06–9.40)	**0.039**	3.2 (1.26–8.00)	**0.014**
N	0-3 vs. ≥4	3.4 (1.08–10.83)	**0.036**	-(-)	-	2.8 (1.09–7.27)	**0.033**	3.1 (1.33–7.33)	**0.009**
Ki-67	<20% vs. ≥20%	1.1 (0.15–8.46)	0.915	-(-)	-	0.9 (0.22–4.06)	0.936	1.6 (0.46–5.21)	0.480
LVI	no vs. yes	0.9 (0.32–2.68)	0.896	-(-)	-	1.2 (0.48–2.96)	0.703	1.27 (0.54–2.97)	0.579
SHH	'low' vs. 'high'	5.2 (0.69–39.78)	0.110	-(-)	-	2.4 (1.00–5.56)	0.051	4.9 (0.66–36.50)	0.119
AR	'low' vs. 'high'	10.0 (1.31–75.99)	**0.026**	10.9 (1.43–83.67)	**0.021**	7.6 (0.98–54.82)	0.052	6.2 (0.84–46.10)	0.074

AR – androgen receptor; CI – confidence interval; ER – estrogen receptor; HR – hazard ratio; LVI – lymphovascular invasion; N – number of positive lymph nodes; *p* – *p*-value; PR – progesterone receptor; SHH – sonic hedgehog protein; T – tumor size; TNBC – triple-negative breast cancer

Supplemental Table S4. Cox proportional hazards regression model analysis of metastasis-free survival. Multivariate analysis was performed only if SHH or AR expression showed statistically significant *p*-value in univariate analysis. Significant *p*-values are in bold.

		All patients				TNBC		ER-	
		Univariate		Multivariate		Univariate		Univariate	
**Covariate**	**Categories**	**HR (95% CI)**	***p***	**HR (95% CI)**	***p***	**HR (95% CI)**	***p***	**HR (95% CI)**	***p***
Age	≤50 vs. >50 years	1.9 (0.57–6.66)	0.292	-(-)	-	5.3 (0.67–41.25)	0.113	4.8 (0.62–36.68)	0.134
T	<2 vs. ≥2 cm	5.5 (1.83–16.66)	**0.002**	4.4 (1.44–13.54)	**0.010**	6.6 (0.85–51.80)	0.072	9.1 (1.19–70.24)	**0.034**
N	0-3 vs. ≥4	5.6 (2.26–13.99)	**0.0002**	4.2 (1.64–10.56)	**0.003**	5.2 (1.52–18.07)	**0.009**	4.2 (1.37–12.92)	**0.012**
Ki-67	<20% vs. ≥20%	8.00 (1.06–59.83)	**0.043**	-(-)	-	0.7 (0.09–5.73)	0.766	1.1 (0.14–8.55)	0.918
LVI	no vs. yes	1.6 (0.64–3.90)	0.315	-(-)	-	1.2 (0.34–3.99)	0.804	1.5 (0.47–4.42)	0.518
SHH	'low' vs. 'high'	0.54 (0.21–1.37)	0.194	-(-)	-	3.7 (0.96–13.83)	0.057	2.7 (0.92–8.16)	0.070
AR	'low' vs. 'high'	0.3 (0.12–0.86)	**0.024**	-(-)	-	1.3E+06 (5.45E-218–32.27E+228)	0.957	1.1E+06 (3.26E-233–36.37E+243)	0.960

		ER+		PR-	
		Univariate		Univariate	
**Covariate**	**Categories**	**HR (95% CI)**	***p***	**HR (95% CI)**	***p***
Age	≤50 vs. >50 years	0.7 (0.13–3.74)	0.661	2.3 (0.51–10.16)	0.282
T	<2 vs. ≥2 cm	2.1 (0.42–10.40)	0.365	6.2 (1.39–27.84)	**0.017**
N	0-3 vs. ≥4	7.9 (1.60–39.36)	**0.011**	4.5 (1.56–13.07)	**0.006**
Ki-67	<20% vs. ≥20%	5.32E+05 (2.77E-172–1.022E+183)	0.950	2.7 (0.35–20.31)	0.346
LVI	no vs. yes	3.1 (0.58–17.10)	0.188	1.4 (0.45–4.04)	0.589
SHH	'low' vs. 'high'	0.2 (0.02–1.28)	0.083	3.1 (0.97–9.91)	0.056
AR	'low' vs. 'high'	0.3 (0.07–1.68)	0.185	9.9E+05 (5.46E-250–1.81E+261)	0.963

AR – androgen receptor; CI – confidence interval; ER – estrogen receptor; HR – hazard ratio; LVI – lymphovascular invasion; N – number of positive lymph nodes; *p* – *p*-value; PR – progesterone receptor; SHH – sonic hedgehog protein; T – tumor size; TNBC – triple-negative breast cancer
